# Susceptibility of Dermatophytes to Thiabendazole Using CLSI Broth Macrodilution

**DOI:** 10.5402/2012/351842

**Published:** 2012-09-13

**Authors:** Efrén Robledo-Leal, Mariana Elizondo-Zertuche, Gloria M. González

**Affiliations:** ^1^Departamento de Microbiología e Inmunología, Facultad de Ciencias Biológicas, Universidad Autónoma de Nuevo León, 66450 San Nicolás de los Garza, NL, Mexico; ^2^Departamento de Microbiología, Facultad de Medicina, Universidad Autónoma de Nuevo León, Madero y Dr. E. A. Pequeño s/n, Colonia Mitras Centro, 64460 Monterrey, NL, México, Mexico

## Abstract

*Objective*. To evaluate *in vitro* antifungal activity of thiabendazole against strains of dermatophytes using a reference method for filamentous fungi. *Materials and Methods*. Dermatophytes' susceptibility to thiabendazole (TBZ) and fluconazole (FCZ) was evaluated using macrodilution method of protocol M38-A2 of the Clinical and Laboratory Standards Institute (CLSI). *Results*. MIC ranges of TBZ for all strains were narrower and/or smaller than those of FCZ. TBZ showed a significantly greater potency than FCZ (*P* = 0.05) against all isolates. *Discussion*. Although there have been approaches to evaluate the antifungal activity of TBZ in human mycoses, no tests had been made with a standardized protocol. Susceptibility data resulted from this study shows that although TBZ is not a particularly strong inhibitor of dermatophytes, it displays a stable and constant effect against all isolates tested. *Conclusion*. Results show that TBZ is more effective against strains of dermatophytes than FCZ. We acknowledge the antifungal effect of TBZ against dermatophyte isolates.

## 1. Introduction

Thiabendazole (TBZ) is a systemic benzimidazole fungicide used to control fruit and vegetables diseases such as mold, rot, blight, and stain. In livestock, thiabendazole is applied as an antihelmintic (PubChem SID: 24900571). Thiabendazole, 2-(4′-thiazolyl) benzimidazole, was first described in 1961 as a broad spectrum antihelmintic [1]; it has been used as a topical treatment against human skin diseases caused by fungi, such as dermatophytosis [2, 3] and chromomycosis [4, 5], and there is also a case report of keratitis due to *Aspergillus flavus* successfully treated with TBZ [6]; it has low acute toxicity (category III) and is neither irritating to the eyes or skin nor is a dermal sensitizer [7]. Although TBZ has been tested against dermatophytes before [8, 9], we could not find any reports where TBZ was submitted to susceptibility testing using the Clinical and Laboratory Standards Institute's protocol M38-A2. The aim of this study was to evaluate the *in vitro *activity of TBZ in comparison to that of fluconazole (FLC).

## 2. Materials and Methods

### 2.1. Dermatophyte Strains

The dermatophyte strains were obtained from Hospital Universitario at Universidad Autónoma de Nuevo León. We used five different species of dermatophytes: *Trichophyton mentagrophytes *(*n* = 10),* T. rubrum *(*n* = 10),* T. tonsurans* (*n* = 10), *Epidermophyton floccosum* (*n* = 5), and *Microsporum canis* (*n* = 10). The isolates were stored as suspensions in water at room temperature until used in the study.

### 2.2. Susceptibility Assay

Protocol M38-A2 of the Clinical and Laboratory Standards Institute (CLSI) was employed [10]. Prior to testing, each isolate was subcultured onto potato dextrose agar (PDA) at 30°C for 7 to 10 days to ensure purity and reactivate metabolic activity. TBZ (Sigma-Aldrich, St. Louis, MO) and FLC (Pfizer Inc., New York, NY) were obtained as reagent grade powders from their respective manufacturers. TBZ dilutions were prepared in polyethylene glycol (PEG). Aliquots (0.1 mL) of each antifungal agent were dispensed in glass test tubes which were stored at −70°C until used. The final concentration of both drugs ranged from 0.125 to 64 *μ*gmL^−1^. Inoculum suspensions were prepared from 5- to 7-day-old cultures grown on PDA at 30°C, except for *T. rubrum*, which was grown on potato dextrose agar mixed with oats extract in order to stimulate sporulation. Inocula were prepared counting conidia in a hemocytometer and adjusting concentration afterwards in RPMI-1640. Each test tube containing the drug aliquots was inoculated with 0.9 mL of the diluted suspensions. Tubes were added for positive growth control and vehicle toxicity in the case of TBZ. The tubes were incubated at 35°C and read 5 and 7 days after incubation. The minimum inhibitory concentration (MIC) was defined as the lowest concentration showing 80% growth inhibition compared to the growth control tube. *Candida parapsilosis *ATCC 22019 and *Candida krusei* ATCC 6258 were included as quality controls and protocol M27-A3 [11] against FLC was performed on them every time a set of isolates was evaluated.

## 3. Results

Table 1 summarizes the *in vitro* susceptibility of TBZ and FCZ. The MIC ranges of TBZ for each strain were narrower and/or smaller than those of FCZ. With the exception of *M. canis*, MICs at which 50% (MIC_50_) and 90% (MIC_90_) of the isolates were inhibited remained the same in all cases for TBZ while for FCZ the MIC_90_ for *T. rubrum, T. mentagrophytes, *and* E. floccosum* doubled the MIC_50_. On average, *Microsporum canis* showed the lowest MIC values for both drugs while *Trichophyton tonsurans* showed the highest. No particular activity was exerted by TBZ to the isolates tested. According to the Mann-Whitney *U* test, TBZ showed a significantly greater potency than FCZ (*P* = 0.05) against all isolates.

## 4. Discussion

TBZ was first registered as a pesticide in the US in 1969 by Merck and has been used since then to control a variety of vegetable diseases caused by various fungi; hence its antifungal effect is not a novel fact. Nevertheless and although there have been approaches to evaluate its antifungal activity in human mycoses, no tests had been made with a standardized protocol. Second edition of CLSI reference method for antifungal susceptibility testing of filamentous fungi now addresses dermatophyte susceptibility guidelines only for the microdilution method, so we followed some of the previously reported procedures [12, 13] in order to apply the macrodilution methodology for this study. We found that oatmeal agar induces abundant sporulation in *T. rubrum*, but the colony growth was slowed down (data not shown), thus not having enough biomass from which to obtain conidia and making it necessary to let the cultures grow for more days or culturing more plates. To solve this issue we used a 1 : 1 mixture of PDA and oatmeal agar in order to maintain the colony growth speed while inducing abundant sporulation. We found that no more than 10 days were enough to obtain the desired inocula with only two plates of *T. rubrum*. 

Susceptibility data resulted from this study shows that although TBZ is not a particularly strong inhibitor of dermatophytes, it displays a stable and constant effect against all isolates tested. This property along with its antifungal large spectrum make it a suitable option for skin mycoses.

## 5. Conclusion

To our knowledge, this is the first study that performs an *in vitro* susceptibility test to TBZ according to the CLSI guidelines. The only other report we acknowledge where TBZ was tested against dermatophytes was that of Battistini et al. published in 1974 and did not follow an *in vitro* standard guideline. Furthermore, they claimed that the effectiveness of TBZ in a polyethylene glycol vehicle may have “a negligible effect”, but the results of our study show otherwise. While this study does not attempt to promote TBZ as the best alternative in the treatment of dermatophyte infections, we suggest the reevaluation of this molecule for its clinical application.

## Figures and Tables

**Table 1:** MICs of 5 different species of dermatophytes to TBZ and FCZ.

Strain (*n*)	MIC (*μ*g/mL) of:											
	Thiabendazole						Fluconazole					
	5 days			7 days			5 days			7 days		
	Range	50%	90%	Range	50%	90%	Range	50%	90%	Range	50%	90%
*T. rubrum* (10)	1	1	1	1-2	2	2	0.5–16	2	4	1–64	4	8
*T. mentagrophytes* (10)	0.25–0.5	0.5	0.5	0.5–1	1	1	8–32	8	16	16–4	16	32
*T. tonsurans* (10)	1	1	1	2	2	2	8–16	16	16	32	32	32
*M. canis* (10)	0.5–1	0.5	1	1	1	1	2–4	4	4	4–8	4	8
*E. floccosum* (5)	1	1	1	1	1	1	8–16	8	16	8–16	8	16

## References

[1] Brown HD, Matzuk AR, Ilves IR (1961). Antiparasitic drugs. IV. 2-(4′-thiazolyl)-benzimidazole, a new anthelmintic. *Journal of the American Chemical Society*.

[2] Battistini F, Zaias N, Sierra R, Rebell G (1974). Clinical antifungal activity of thiabendazole. *Archives of Dermatology*.

[3] Wallace SM, Shah VP, Epstein WL, Greenberg J, Riegelman S (1977). Topically applied antifungal agents. Percutaneous penetration and prophylactic activity against *Trichophyton mentagrophytes* infection. *Archives of Dermatology*.

[4] de Rosanda MA (1979). Susceptibility tests of *Fonsecaeapedrosoi* strains to the combination ofthiabendazole and amphotericin B *in vitro*. *Revista do Instituto de Medicina Tropical dec São Paulo*.

[5] De Clerq D, Kakiesse M, De Vroey C, Mazebo P (1985). Treatment of chromomycosis 14 with a combination of 5-fluorocytosine and thiabendazole. Apropos of a Zairian case 15 dueto *Fonsecaeapedrosoi*. *Annales de la Societe Belge de Medecine Tropicale*.

[6] Madan PW, Edna PW, Achyut PS (1980). Keratitis due to *Aspergillusflavus* successfully treated with thiabendazole. *The British Journal of Ophthalmology*.

[7] Blaszcak, Thiabendazole D (Batch #Dr M6 17): Acute Dermal ToxicityStudy in Rabbits: 21 Project ID, 4004-86.

[8] Robinson HJ, Phares H, Graessle OE (1964). Antimycotic properties of thiabendazole. *The Journal of Investigative Dermatology*.

[9] Blank H, Rebell G (1965). Thiabendazole activity against the fungi of dermatophytosismycetomas and chromomycosis. *The Journal of Investigative Dermatology*.

[12] Barros MEDS, Santos DDA, Hamdan JS (2006). In vitro methods for antifungal susceptibility testing of Trichophyton spp. *Mycological Research*.

[13] Siqueira ER, Ferreira JC, Pedroso RDS, Lavrador MAS, Candido RC (2008). Dermatophyte susceptibilities to antifungal azole agents tested in vitro by broth macro and microdilution methods. *Revista do Instituto de Medicina Tropical de Sao Paulo*.

